# BMI1 and Mel-18 oppositely regulate carcinogenesis and progression of gastric cancer

**DOI:** 10.1186/1476-4598-9-40

**Published:** 2010-02-21

**Authors:** Xiao-Wei Zhang, Ya-Ping Sheng, Qian Li, Wei Qin, You-Wei Lu, Yu-Fan Cheng, Bing-Ya Liu, Feng-Chun Zhang, Jin Li, Goberdhan P Dimri, Wei-Jian Guo

**Affiliations:** 1 Department of Medical Oncology, Cancer Hospital of Fudan University, 270 Dong An Road, Shanghai 200032, China; 2 Department of Oncology, Shanghai Medical College, Fudan University, Shanghai, China; 3 Department of Medical Oncology, Xinhua Hospital, School of Medicine, Shanghai Jiaotong University, Shanghai 200092, China; 4 Department of Medical Oncology, Renji Hospital, School of Medicine, Shanghai Jiaotong University, Shanghai 200001, China; 5 Shanghai institute of digestive surgery, Shanghai 200025, China; 6 Department of Medicine, NorthShore University HealthSystem Research Institute, 1001 University Place, Evanston, IL 60201, USA

## Abstract

**Background:**

The *BMI1 *oncogene is overexpressed in several human malignancies including gastric cancer. In addition to BMI1, mammalian cells also express Mel-18, which is closely related to BMI1. We have reported that Mel-18 functions as a potential tumor suppressor by repressing the expression of BMI1 and consequent downregulation of activated AKT in breast cancer cells. However, the mechanisms of BMI1 overexpression and the role of Mel-18 in other cancers are still not clear. The purpose of this study is to investigate the role of BMI1 and Mel-18 in gastric cancer.

**Results:**

BMI1 was found to be overexpressed in gastric cancer cell lines and gastric tumors. Overexpression of BMI1 correlated with advanced clinical stage and lymph node metastasis; while the expression of Mel-18 negatively correlated with BMI1. BMI1 but not Mel-18 was found to be an independent prognostic factor. Downregulation of BMI1 by Mel-18 overexpression or knockdown of BMI1 expression in gastric cancer cell lines led to upregulation of p16 (p16INK4a or CDKN2A) in p16 positive cell lines and reduction of phospho-AKT in both p16-positive and p16-negative cell lines. Downregulation of BMI1 was also accompanied by decreased transformed phenotype and migration in both p16- positive and p16-negative gastric cancer cell lines.

**Conclusions:**

In the context of gastric cancer, *BMI1 *acts as an oncogene and Mel-18 functions as a tumor suppressor via downregulation of BMI1. Mel-18 and BMI1 may regulate tumorigenesis, cell migration and cancer metastasis via both p16- and AKT-dependent growth regulatory pathways.

## Background

Polycomb group (PcG) proteins are evolutionary conserved gene silencers, which play an important role in the development of vertebrate organisms. These proteins regulate cell proliferation, senescence and tumorigenesis via well-known growth regulatory pathways [1]. Overexpression of key PcG proteins such as BMI1 has been found in several human malignancies including breast cancer, colorectal cancer, nasopharyngeal carcinoma, melanoma, gastric cancer, and bladder cancer [2-7]. Overexpression of BMI1 often correlates with poorer prognosis [4,6,7]. BMI1 also plays an important role in self-renewal of hematopoietic stem cells (HSCs), neural stem cells and mammary stem cells [8-10].

After a finite number of cell divisions, most normal human cells undergo cellular senescence, which constitutes a powerful barrier to oncogenesis[11]. Overexpression of BMI1 has been shown to bypass this barrier in human mammary epithelial cells and fibroblasts [12,13]. In addition to BMI1, mammalian cells also express a BMI1-related PcG protein Mel-18 (also known as polycomb group ring finger 2 (PCGF2)). The Mel-18 gene product is structurally highly similar to BMI1 protein. Interestingly, we have found that BMI1 is negatively regulated by Mel-18 and that Mel-18 is overexpressed in senescent fibroblasts. Accordingly, Mel-18 overexpression leads to accelerated or premature senescence in proliferating fibroblasts by repression of BMI1 [14]. Similar to human fibroblasts, expression of Mel-18 negatively correlates with BMI1 in a number of breast cancer cell lines and breast tumors [15]. Negative correlation between BMI1 and Mel-18 expression was also recently reported in hematopoietic stem cells [16].

We also reported that Mel-18 overexpression in breast cancer cell line MCF7 results in downregulation of BMI1 and reduction of transformed phenotype. Furthermore, downregulation of BMI1 by Mel-18 overexpression and knockdown of BMI1 expression by RNA interference (RNAi) approach is accompanied by downregulation of AKT/PKB (Protein Kinase B) activity [15]. Thus our data suggested that Mel-18 acts as a tumor suppressor in breast epithelial cells. Consistent with our data, Lee et al. also recently reported that Mel-18 negatively regulates AKT and that its overexpression inhibits growth of breast cancer cells [17]. However, the function of Mel-18 is still debatable. It is described as a potential tumor suppressor in some studies [17-19]; while in other studies, it was found that similar to *BMI1*, *Mel-18 *can act as an oncogene [20,21]. The opposite role of BMI1 and Mel-18 in human fibroblasts and breast cancer cells is an interesting finding. However, it needs to be verified in other cell types and pathological conditions. Importantly, the role of Mel-18 in cancers other than breast and prostate cancers is still not clear. Regulation of AKT/PKB pathway by BMI1 and Mel-18 activity also needs further confirmation.

Gastric cancer is one of the most common malignancies throughout the world, and mechanisms that underlie the carcinogenesis of gastric cancer are still poorly understood. It has been reported that BMI1 is overexpressed in gastric cancer cells and is an independent prognosis factor [6]. However, the exact role of BMI1 in gastric cancer is far from clear. Potential tumor suppressive role of Mel-18 in gastric cancer is also not known. Here, we show that BMI1 is overexpressed in gastric cancer cell lines and gastric tumors, and its expression correlated with advanced clinical stage, lymph node metastasis, and poor prognosis. Importantly, we show that expression of Mel-18 is decreased in gastric cancer and is negatively correlated with the expression of BMI1 in both gastric cancer cells and normal gastric epithelial cells. We also report that downregulation of BMI1 by Mel-18 overexpression and knockdown of BMI1 expression in gastric cell lines is accompanied by downregulation of AKT/PKB activity and upregulation of p16, which resulted in induction of a senescence-like phenotype and reduction of transformed properties in gastric cancer cell lines. These data suggest that *BMI1 *acts as an oncogene via regulation of p16 and AKT/PKB, and *Mel-18 *acts as a tumor suppressor via downregulation of BMI1 during the development of gastric cancer.

## Methods

### Cellular and molecular reagents, and methods

An immortalized human gastric mucosal epithelial cell line (GES-1) and eight human gastric cancer cell lines (MKN28, MKN45, KATOIII, NCI-N87, SUN-1, SUN-16, SGC-7901 and AGS) were obtained from the Surgical Institution of Ruijin Hospital. These cell lines were cultured in RPMI-1640 supplemented with 10% fetal bovine serum (FBS) and antibiotics. Stable cell lines expressing *Mel-18 *or other genes of interest were generated by infection of the retroviral vectors expressing the particular genes as described [22]. Retroviral vectors overexpressing Mel-18, BMI1 shRNA (BMI1 i) and a control shRNA (Ctrl i) are described earlier [14]. The retroviruses were produced by transient transfection of the retroviral vector together with pIK packaging plasmid into 293 packaging cell line as described [22]. Induction of senescence in cancer cells was determined using Senescence-associated beta galactosidase (SA-β-gal) assay as described [12], and the apoptosis was determined using Annexin -FITC Apoptosis Detection Kit (BD Biosciences, USA) as described [23]. Soft-agar assay, wound healing assay and transwell chamber (Corning Costar, Cambridge, MA) migration assays were done as described [24].

### Clinical samples

Seventy five paraffin-embedded gastric cancer tissue samples and twenty two normal gastric mucosal tissue samples were obtained from the archives of the Department of Pathology for further immunohistochemical analysis of different proteins expression. The clinicopathologic variables and survival data were obtained from the medical records and the disease stages of the patients were classified according to the 2002 UICC gastric cancer TNM staging system. Prior patients' consent and approval from the Institute Research Ethics Committee were obtained for the use of clinical materials described in the present study.

### Quantitative real time RT-PCR (QRT-PCR) assays

The QRT-PCR was carried out as described [14] using Brilliant SYBR Green QRT-PCR Master Mix, 2-Step kit (Stratagene, La Jolla, CA). The PCR amplification was carried out using PTC-200 Real Time PCR system (MJ Research Inc, USA). The primers for QRT-PCR were- *GAPDH *forward (F)-5' GCTGAACGGGAAGCTCACTG 3', *GAPDH *reverse (R) - 5'GTGCTCAGTGTAGCCCAGGA3'; *BMI1 *F 5' GCTTCAAGATGGCCGCTTG 3', *BMI1 *R 5' TTCTCGTTGTTCGATGCATTTC 3'; and *Mel-18 *F 5' GATGGATGTGCC CAGCAAGT 3', *Mel-18 *R 5' GGAGCCTTGT CGCTGACTGA 3'. The Ct (threshold cycle) value of *Mel-18 *or *BMI1 *amplification was normalized to that of *GAPDH *control. The mRNA levels of target genes in gastric cancer cell lines were compared with that in the immortalized human normal gastric epithelial cell line GES-1, and when the expression showed a 2-fold increase or decrease, it was considered as an altered expression.

### Immunological reagents, Western blot, and Immunohistochemical analyses

BMI1 was detected using F6 mouse monoclonal antibody (mAb) from Upstate Cell Signaling Solutions (Charlottesville, Virginia). Mel-18 was detected by a rabbit polyclonal H-115 (Santa Cruz Biotech., CA). The 9E10 mAb (Santa Cruz Biotech., CA) against c-Myc was used to detect the expression of c-Myc tag in exogenously expressed protein Mel-18. Antibodies against human total AKT (tAKT) and phospho-AKT (ser-473, pAKT) were obtained from Cell Signaling Technology (Beverly, MA). Western blot analysis to detect the altered expression of BMI1, Mel-18, and other proteins in gastric cells were performed as described [22]. Immunohistochemical (IHC) analysis to detect the expression of Mel-18, BMI1, pAKT, and p16 in paraffin sections was performed as described [4]. All slides were interpreted by two independent observers in a blinded fashion. When more than 10% of the cells in a sample were stained with moderate to strong staining, the sample was considered positive. Otherwise, the samples were considered negative.

### Statistical analysis

All statistical analyses were done by using the SPSS 15.0 software package. Two-tailed P value less than 0.05 was considered statistically significant. In the set of IHC assay of paraffin-embedded tissue samples, the Pearson χ^2 ^test was used to determine the correlation between the expression of Mel-18 or BMI1 and other proteins, and clinicopathologic characteristics. Cumulative survival curves were plotted by the Kaplan-Meier method and the relationship between each of the variables and survival was assessed by Log-rank test in a univariate analysis. The parameters were then tested by multivariate Cox proportional hazards model, which was performed to identify independent variables for predicting survival. In *in vitro *experiments, data were described as mean ± SD, and analyzed by Student's t-test.

## Results

### Expression of BMI1 and Mel-18 inversely correlates in gastric cancer cell lines and gastric tumors

**Figure 1:**
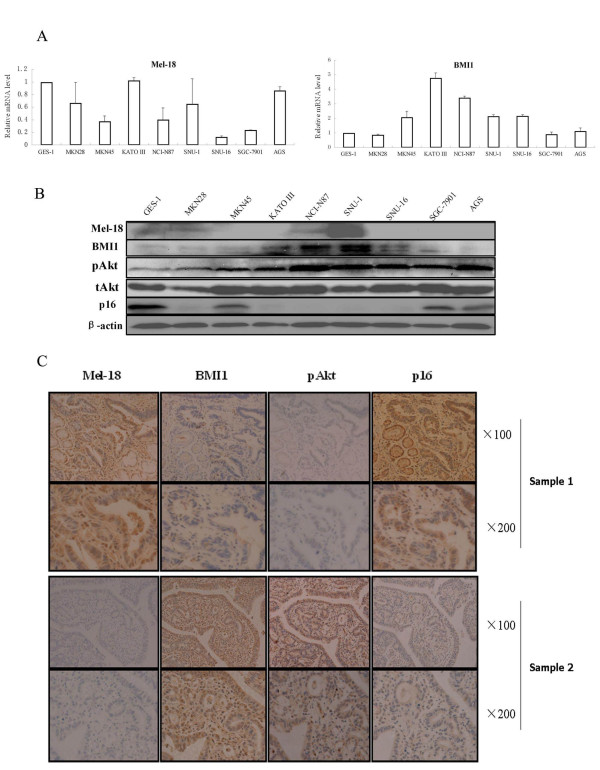
**Mel-18 and BMI1 expression inversely correlates in gastric cancer cell lines and gastric tumors**. **A) **Mel-18 (left) and BMI1 (right) expression in an immortalized human normal gastric epithelial cell line GES-1 and various gastric cancer cell lines as detected by QRT-PCR analysis. The bars indicate mean ± SD from three different experiments. **B) **BMI1, Mel-18, phospho-AKT (pAKT), total AKT (tAKT), and p16 proteins expression in an immortalized human normal gastric epithelial cell line GES-1 and various gastric cancer cell lines as detected by Western blot analysis. β-actin was used as a loading control. **C**) A representative figure of two gastric tumor samples. Sample 1 expresses high Mel-18, low BMI1, low pAKT, and high p16, while the second sample expresses low Mel-18, high BMI1, high pAKT, and low p16. Tissue sections were stained with BMI1, Mel-18-, pAKT-, or p16- specific antibodies and counterstained with hematoxylin as described in experimental procedures.

Our previous data showed an inverse correlation between BMI1 and Mel-18 expression in cultured human fibroblasts and breast cancer cells. Based on these data, we hypothesized that gastric cancer cell lines and gastric tumors may also express high BMI1 and low Mel-18. To probe this hypothesis, first we analyzed the expression of BMI1 and Mel-18 in several gastric cancer cell lines. Our results showed that compared to GES-1, a normal immortal human gastric mucosal epithelial cell line, the majority of gastric cancer cell lines expressed high BMI1 (5 out of 8 at the RNA and protein levels) and low Mel-18 (4 out of 8 at the RNA level and 5 out of 8 at the protein level) (Fig 1A, 1B).

Next, we studied the expression of Mel-18 and BMI1 in gastric tumors by IHC. IHC analysis of paraffin-embedded archival gastric tumor samples showed that 51 of 75 (83.6%) samples exhibited positive staining for BMI1 while only one sample out of 21 normal gastric mucosal tissue samples (1/21, 4.8%) was scored positive for BMI1. Furthermore, 15 of 75 (24.5%) of the gastric tumor samples and 12 of 21 (57.1%) of the normal gastric mucosal tissue samples showed a positive staining for Mel-18. Compared with normal gastric mucosal tissues, gastric tumor tissues expressed significantly higher BMI1 and lower Mel-18 (Table 1). The correlation analysis between BMI1 and Mel-18 proteins expression also showed a strong negative correlation in the gastric tumors and normal gastric mucosal tissues (P = 0.002, Table 1, Fig 1C).

**Table 1:** The expression of Mel-18, BMI1, pAkt, and p16; and the correlations between these proteins in gastric cancer tissues and normal gastric mucosa tissues.

		Mel-18 n (%)			BMI1 n (%)			pAkt n (%)			p16 n (%)		
	
		(-)	(+)	P value	(-)	(+)	P value	(-)	(+)	P value	(-)	(+)	P value
**Tissues**													
Normal mucosa		9(42.9)	12(57.1)		20(95.2)	1(4.8)		18(85.7)	3(14.3)		6(28.6)	15(71.4)	
Gastric cancer		59(78.7)	16(21.3)	0.001*	32(42.7)	43(57.3)	<.0001*	33(44.0)	42(56.0)	0.0007*	32(42.7)	43(57.3)	0.243
**Proteins**													
**Bmi-1**													
Negative (-)		29(56.9)	22(43.1)										
Positive (+)		38(86.4)	6(13.7)	0.002*									
**pAkt**													
Negative (-)		36(72.0)	14(28.0)		35(70.0)	15(30.0)							
Positive (+)		32(69.6)	14(30.4)	0.793	17(37.0)	29(63.0)	0.0012*						
**p16**													
Negative (-)		31(81.6)	7(18.4)		11(28.9)	27(71.1)		18(47.4)	20(52.6)				
Positive (+)		37(63.8)	21(36.2)	0.061	41(70.7)	17(29.3)	<.0001*	33(56.9)	25(43.1)	0.360			

*Data were analyzed by the χ2-test and p < 0.05 was considered to be significant

### Overexpression of BMI1 correlates with high phosphorylated AKT and low p16

We have previously reported that p16 and AKT are potential cancer-relevant targets of BMI1 and Mel-18 in human fibroblasts and breast cancer cells respectively. To determine whether BMI1 and Mel-18 regulate p16 and AKT during gastric carcinogenesis, we analyzed the expression of p16 and phosphorylated AKT in gastric cancer cell lines and gastric cancer tissues. We found that the normal immortal human gastric mucosal epithelial cell line GES-1 expressed high levels of p16; while 3 of 8 gastric cancer cell lines expressed lower levels of p16, and 5 of 8 gastric cancer cell lines did not express any detectable p16. Importantly, cell lines with low or no p16 overexpressed BMI1 suggesting a negative correlation between the expression of BMI1 and p16 (Fig 1B). Compared to GES-1, all of the gastric cancer cell lines expressed high level of phosphorylated AKT. Thus the overexpression of BMI1 correlated with high AKT activity and low p16 in gastric cancer cell lines (Fig 1B).

Next, we carried out IHC analysis of archival paraffin-embedded gastric tumor biopsies. The results showed that 43 of 75 (57.3%) tumor biopsies and 15 of 21 (71.4%) normal gastric mucosal tissue samples stained positively for p16. On the other hand, 56.0% (42 of 75) of gastric cancer tissues and 14.3% (3 of 21) of normal gastric mucosal tissues stained positively for phospho-AKT. Statistical analysis of data showed that compared to normal gastric mucosal tissues, gastric tumors expressed significantly high phosphorylated AKT and low levels (but not significant) of p16 (Table 1). The correlation between Mel-18 and p16 or phosphorylated AKT was also not significant. However, correlation analysis showed that the overexpression of BMI1 negatively correlated with low level of p16, and positively correlated with high phospho-AKT (Table 1, Fig 1C).

### The correlation between the expression of BMI1 and Mel-18 with clinicopathologic characteristics and prognosis

**Figure 2:**
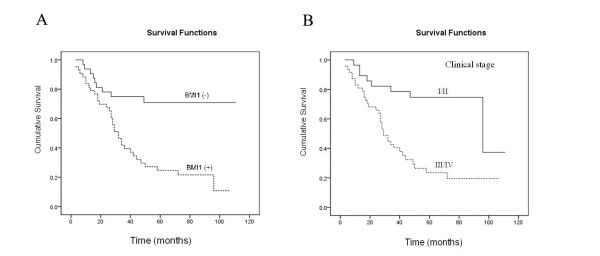
**BMI1 is an independent prognostic marker in gastric cancer patients**. **A) **Kaplan-Meyer survival curves were plotted as cumulative survival vs months according to BMI1 expression (negative and positive). **B) **Kaplan-Meier survival curves were plotted according to clinical stage (stage I/II and stage III/IV).

There was a significant positive correlation between BMI1 expression with lymph node metastasis, or clinical stage in paraffin-embedded archival gastric tumor samples as the expression of BMI1 was significantly higher in the patients with positive lymph node metastasis, or late stage disease (Table 2). Overexpression of phospho-AKT also positively correlated with depth of invasion (T classification), or lymph node metastasis (N classification), and the expression of p16 negatively correlated with lymph node metastasis (Table 2). Multivariate Cox proportional hazards model analysis, which included gender, age, tumor size, histology, T classification, lymph node metastasis, distant metastasis, clinical stage, and the expression of Mel-18, BMI1, phospho-AKT and p16 proteins, showed that BMI1 protein expression, and clinical stage were independent prognostic indicators of overall survival, while Mel-18 was not the independent prognostic indicator. The 5-year survival rate in the BMI1 positive group was significantly lower than that in the BMI1 negative group (23.3% vs. 50.0%, p < 0.001. Fig 2). These results suggest that overexpression of BMI1 correlates with the poor prognosis of patients with gastric cancer.

**Table 2:** Correlations between the expression of BMI1, Mel-18, pAkt, p16 proteins and clinicopathologic variables

Variables		Mel-18 n (%)			BMI1 n (%)			pAkt n (%)			p16 n (%)		
								
		(-)	(+)	P value	(-)	(+)	P value	(-)	(+)	P value	(-)	(+)	P value
**Gender**													
Male		40(80)	10(20)		21(42)	29(58)		21(42)	29(58)		20(40)	30(60)	
Female		19(76)	6(24)	0.6902	11(44)	14(56)	0.8689	12(48)	13(52)	0.6217	12(48)	13(52)	0.509
**Age (years)**													
<60		27(84.4)	5(15.6)		14(43.8)	18(56.2)		17(53.1)	15(46.9)		15(46.9)	17(53.1)	
≥60		32(74.4)	11(25.6)	0.2979	18(41.9)	25(58.1)	0.87	16(37.2)	27(62.8)	0.1696	17(39.5)	26(60.5)	0.525
**Size(cm)**													
<4.5		34(81.0)	8(19.0)		18(42.9)	24(57.1)		20(47.6)	22(52.4)		18(42.9)	24(57.1)	
≥4.5		25(75.8)	8(24.2)	0.5857	14(42.4)	19(57.6)	0.97	13(39.4)	20(60.6)	0.4763	14(42.4)	19(57.6)	0.6177
**Histology**													
Well differentiated		23(74.2)	8(25.8)		14(45.2)	17(54.8)		13(41.9)	18(58.1)		12(38.7)	19(61.3)	
Poorly differentiated		36(81.8)	8(18.2)	0.4274	18(40.9)	26(59.1)	0.7139	20(45.5)	24(54.5)	0.7139	20(45.5)	24(54.5)	0.5609
**T classification**													
T1/2		21(84)	4(16)		13(52)	12(48)		15(60)	10(40)		9(36)	16(64)	
T3/4		38(76)	12(24)	0.4253	19(38)	31(62)	0.2478	18(36)	32(72)	0.0484*	23(46)	27(54)	0.4091
**LNM**													
Negative		33(80.5)	8(19.5)		25(61.0)	16(39.0)		24(58.5)	17(41.5)		13(31.7)	28(68.3)	
Positive		26(76.5)	8(23.5)	0.6725	7(20.6)	27(79.4)	0.0004*	9(26.5)	25(73.5)	0.0054*	19(55.9)	15(44.1)	0.0351*
**Distant metastasis**													
Negative		55(80.9)	13(19.1)		31(45.6)	37(54.4)		32(47.1)	36(52.9)		29(42.6)	39(57.4)	
Positive		4(57.1)	3(42.9)	0.1443	1(14.3)	6(85.7)	0.1108	1(14.3)	6(85.7)	0.0963	3(42.9)	4(57.1)	0.9915
**Clinical stage**													
I/II		25(86.2)	4(13.8)		18(62.1)	11(37.9)		16(57.1)	12(42.9)		11(39.3)	17(60.7)	
III/IV		34(73.9)	12(26.1)	0.2056	14(30.4)	32(69.6)	0.007*	17(36.2)	30(63.8)	0.0768	21(44.7)	26(55.3)	0.6477

Abbreviations: LNM, lymph node metastases. *Data were analyzed by the χ2-test and p < 0.05was considered to be significant.

### Overexpression of Mel-18 results in down-regulation of BMI1 and induction of senescence in gastric cancer cells

**Figure 3:**
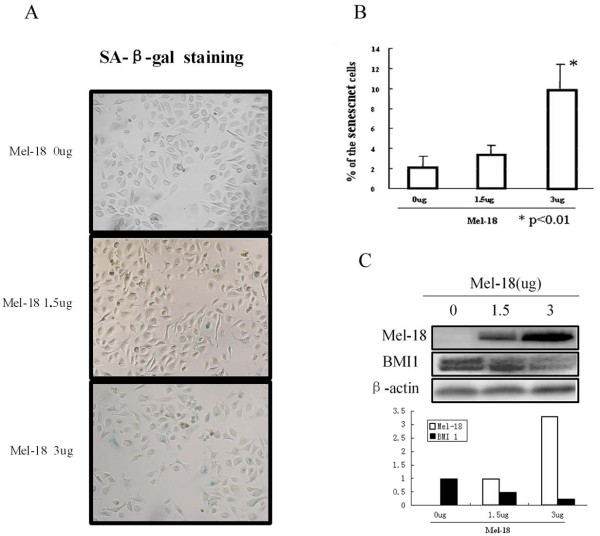
**Transient transfection of Mel-18 in SGC-7901 cells results in the downregulation of BMI1 and increased senescence in a dose-dependent manner**. **A) **Transient transfection of Mel-18 results in increased senescence in a dose-dependent manner as determined by SA-β-gal staining analysis. SGC-791 cells were transfected with 0, 1.5, and 3 μg of Mel-18 overexpressing plasmid and analyzed for the induction of senescence using SA-β-gal assay. **B)** Senescent cells (SA-β-gal positive) from three different experiments were counted and plotted. **C)** Transient transfection of Mel-18 in SGC-7901 cells results in the dose-dependent downregulation of BMI1 as determined by Western blot analysis, β-actin serves as internal control ( upper panel: Mel-18, BMI1, and β-actin were detected by Western blot; lower panel: quantification of the levels of Mel-18 and BMI1. The levels of Mel-18 and BMI1 were quantified by densitometric analysis of signal present in each lane using ImageJ 1.37v software (NIH, Bethesda, USA) and normalized to β-actin signal of each lane, and plotted. ).

**Figure 4:**
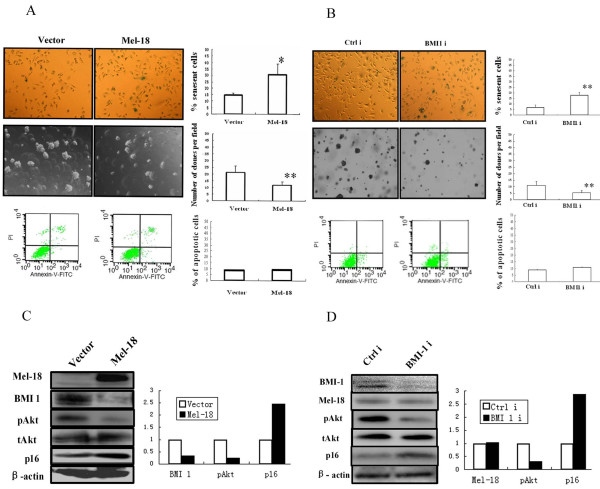
**Reduction of transformed phenotype by Mel-18 overexpression and knockdown of BMI1 expression**. **A) **Overexpression of Mel-18 in SGC-7901 cells results in increased cellular senescence (p < 0.05; upper panel: left, pictures of SA-β-gal stained cells; right, senescent cells were counted and plotted), decreased number of colonies in soft agar (p < 0.01, mid panel: left, pictures of colonies in soft agar, right, the number of colony were counted and plotted); and no difference in apoptosis (lower panel). The bars indicate mean ± SD. Vector infected cells served as a control. **B)** Knockdown of BMI1 in SGC-7901 results in increased cellular senescence (p < 0.05; upper panel: left, pictures of SA-β-gal stained cells, right, senescent cells were counted and plotted); decreased number of colonies in soft agar (p < 0.05; mid panel: left, pictures of colonies in soft agar, right, the number of colony were counted and plotted); and no difference in apoptosis (lower panel). Ctrli (non-specific sequence) served as control for BMI1 knockdown (BMI1i). **C)** Overexpression of Mel-18 leads to downregulation of BMI1, and reduction in pAKT (phospho-AKT) and upregulation of p16 expression as determined by Western blot analysis ( left, Mel-18, BMI1, pAKT, tAKT (total AKT), p16, and β-actin were detected by Western blot; right, quantification of the levels of BMI1 and p16, and AKT activity by densitometric analysis as described in Figure 3 ). **D) **BMI1 knockdown by RNAi approach leads to no change of Mel-18, reduction in pAKT and upregualtion of p16 expression as determined by Western blot analysis (left, pictures of Western blot; right, quantification of the levels of Mel-18 and p16, and AKT activity by densitometric analysis).

As the expression of Mel-18 and BMI1 inversely correlated in gastric cancer cell lines and gastric tumors, we suspected that similar to breast cancer cells, Mel-18 may negatively regulate the expression of BMI1 in gastric cancer cells. To probe this hypothesis, we transiently transfected SGC-7901 cells with a Mel-18 overexpressing plasmid and measured the changes in the expression of BMI1. We found that overexpression of Mel-18 resulted in down-regulation of BMI1 expression in SGC-7901 cells (Fig 3C). To determine if downregulation of BMI1 by Mel-18 overexpression resulted in induction of a senescence-like phenotype, we performed SA-β-gal staining. The results showed that overexpression of Mel-18 indeed induced senescence in SGC-7901 gastric cancer cells (Fig 3A and 3B). Stable cell lines which overexpress Mel-18 also showed a decreased expression of BMI1 in SGC-7901 (Fig 4C), AGC, and NCI-N87 gastric cell lines (data not shown). To determine whether BMI1 can also inversely regulate Mel-18 expression, we stably expressed BMI1 shRNA in SGC-7901 cells, and examined expression of Mel-18. Our results showed an efficient knockdown of BMI1 but no change in expression of Mel-18 suggesting that BMI1 does not regulate Mel-18 expression (Fig. 4D).

### Overexpression of Mel-18, as well as knockdown of BMI1 results in reduction of transformed phenotype in gastric cancer cells

We determined the transformation potential of control and Mel-18 overexpressing SGC-7901 cells using anchorage-independence growth assay, and determined the induction of senescence and apoptosis by SA-β-gal staining and Annexin V staining respectively. The results indicated that Mel-18 overexpression in SGC-7901 cells which express detectable p16 led to an increase in senescence and a decrease in colony formation in soft agar (Fig. 4A). Compared to control, the number of senescent cells was also higher in Mel-18 overexpressing SGC-7901 cells (Fig. 4A upper panel), and the soft agar colonies in Mel-18 overexpressing SGC-7901 cells were less in frequency and also smaller in size (Fig. 4A middle panel). We also determined the senescence and anchorage-independent growth potential of SGC-7901 cells, which stably express BMI1 shRNA. Western blot analysis of BMI1 indicated efficient knockdown of BMI1 expression in these cells (Fig. 4D). Furthermore, stable expression of BMI1 shRNA in SGC-7901 cells led to a significant increase of number of senescent cells and decrease in number of colonies in soft-agar indicating a decrease in transformed phenotype of these cells (Fig. 4B). Annexin V staining suggested no difference in apoptosis between Mel-18 overexpressing cells, BMI1 knock-down cells and control cells (Fig 4A and 4B lower panels), indicating that in gastric cancer cells BMI1 downregulation does not cause significant cell death. We also found that overexpression of Mel-18, as well as knockdown of BMI1 expression induces senescence and reduces malignancy in AGS cells, another gastric cancer cell line which expresses p16, and in NCI-N87 cells which do not express detectable p16 (data not shown). These results showed that overexpression of Mel-18, or knockdown of BMI1 reduces malignancy of both p16 positive and p16-negative gastric cancer cell lines, suggesting that the mechanism of reduction in malignancy by BMI1 downregulation is at least in part independent of p16 pathway.

### Overexpression of Mel-18, as well as knockdown of BMI1 expression inhibits cell migration in gastric cancer cells

**Figure 5:**
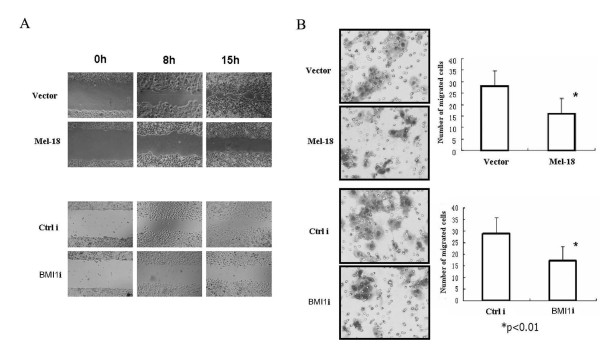
**Mel-18 overexpression as well as BMI1 knockdown in SGC-7901 cells inhibits cell migration**. **A) **Wound healing assays were performed by plating cells in 6-well culture dishes. Cells were scratched in the middle and then further incubated for 8 hrs and 15 hrs, and the wound widths were examined under a light microscope. The migration distance of Mel-18 overexpressed SGC-7901 cells (upper panel), as well as BMI1 knockdown cells (lower panel), was decreased compared with that of control cells. Vector and Ctrl i serve as control for Mel-18 overexpression, and BMI1 knockdown (BMI1i) respectively. **B) **Transwell migration assays using the Corning chamber showed that fewer number of cells migrated in SGC-7901 cells with Mel-18 overexpression or BMI1 knockdown compared with that in control (p < 0.05; left, picture of migrated cells; right, the number of migrated cells were counted and plotted).

Because expression of Mel-18 or BMI1 correlated with lymph node metastasis, we hypothesized that these PcG proteins may regulate cancer metastasis. In support of this hypothesis, we used an *in vitro *cell migration model to measure the effect of Mel-18 and BMI1 on cell migration, which is one of the important steps in cancer metastasis. First, we analyzed the migration distance of scratched cells into the cell-free "scratch" region using a wound healing assay. The results showed that the migration distance of Mel-18 overexpressed SGC-7901 cells, as well as BMI1 knockdown SGC-7901 cells, was decreased significantly compared to the control cells (Fig 5A). Next, we measured the migration potential of cells by Transwell chambers. Results using Transwell chambers also showed that the number of migrated cells decreased significantly in Mel-18 overexpressed SGC-7901 cells, as well as in BMI1 knockdown SGC-7901 cells, compared to that of control cells (Fig 5B). We also determined the effect of Mel-18 overexpression and BMI1 knockdown on migration in NCI-N87 cells which do not express detectable p16. Our data suggested that similar to SGC-7901 cells, BMI1 downregulation in NCI-N87 cells lead to decrease in migration (data not shown). In summary, our results suggest that Mel-18 and BMI1 may regulate cell migration and that BMI1 overexpression may contribute to metastasis. Our data also suggest that regulation of migration by BMI1 and Mel-18 may be independent of p16.

### Mel-18 and BMI1 regulate p16 expression and AKT activity in gastric cancer cell lines

To determine the possible mechanisms of decreased transformed phenotype, and migration inhibition in gastric cancer cell lines by Mel-18 overexpression or knockdown of BMI1 expression, we examined the expression of p16 and phosphorylated AKT in control and Mel-18 overexpressing or BMI1 knockdown cells. Consistent with our previous data that BMI1 and Mel-18 may regulate p16 and AKT, results showed that BMI1 downregulation by Mel-18 overexpression or RNAi approach leads to upregulation of p16 and substantial reduction in phospho-AKT in SGC-7901 cells suggesting that BMI1 regulates AKT activity (Fig. 4C, 4D). In NCI-N87 cells which do not express detectable p16, we also found that overexpression of Mel-18 or knockdown of BMI1 inhibits AKT activity (data not shown).

## Discussion

Arguably BMI1 is the most well studied PcG protein that is known to regulate proliferation and senescence in mammalian cells. It not only inhibits senescence and immortalizes human mammary epithelial cells (HMECs) [13], but it can also transform keratinocytes via downregulation of tumor suppressors and differentiation related factors[25]. It also cooperates with H-Ras to transform HMECs via dysregulation of multiple growth-regulatory pathways [24], and plays a central role in mediating leukemic transformation and development [26]. Aberrant expression of BMI1 has been found in several human cancers and its overexpression is often correlated with poor prognosis in many types of cancers[2-7]. Most importantly, BMI1 is required for self-renewal of normal and malignant stem cells [8-10]. Hence, BMI1 is considered an important therapy target [27,28]. Because BMI1 is an important regulator of cell proliferation and stem cell phenotype, its own regulators are likely to be very important for onocogenesis and self-renewal of normal and cancer stem cells.

We have previously reported that BMI1 is regulated by another PcG protein Mel-18 in human fibroblasts [14], and that by doing so Mel-18 can potentially function as a tumor suppressor. Potential tumor suppressor role of Mel-18 has also been suggested in few other studies [15,17]. In the present study we provide *in vitro *and *in vivo *evidences supporting this notion during gastric cancer development. Overexpression of BMI1 in gastric cancer has been previously reported [6,29], however the potential mechanism of its overexpression remained unclear. Here we confirmed previous observation that BMI1 is overexpressed in gastric cancer cell lines and gastric tumors and that BMI1 overexpression correlates with poor prognosis.

Importantly, here we show that Mel-18 is downregulated in gastric cancer cells and gastric tumors, and that there is a negative correlation between Mel-18 and BMI1 expression in gastric cancer cells. Thus, our data suggest that overexpression of BMI1 in gastric tumors and cell lines may due to downregulation of Mel-18 in gastric cancer cells. Consistent with its tumor suppressor role, we also found that Mel-18 negatively regulated AKT expression, and induced p16 expression and senescence in gastric cancer cell lines. In a recent study, BMI1 overexpression was closely related with the Lauren's and Borrmann's classification and clinical stage in gastric tumors [29]. However, in our study we did not find correlation between BMI1 expression and tumor size, T classification or differentiation in gastric tumors, which was not consistent with the *in vitro *study that BMI1 regulates proliferation. The discrepancy could be due to the limited number of samples in our study or other interfering factors. However, we did find that BMI1 overexpression positively correlated with lymph node metastasis, and clinical stages of the tumors. Our *in vitro *study also showed that overexpression of Mel-18, and knockdown of BMI1 expression, inhibit the ability of migration in gastric cancer cells. It's the first time to find that Mel-18 and Bmi-1 regulate cellular migration in *in vitro *model, and provide preliminary direct evidence for the possibility of Mel-18 and Bmi-1 regulate the metastasis of cancer. Collectively, our data suggest that Mel-18 and BMI1 not only play important roles in tumorigenesis, but may also involve in the progression and metastasis of gastric cancer.

It is interesting to note that our data suggest that BMI1 but not the Mel-18 is an independent negative prognosis factor. Patients with high BMI1 expression survived significantly shorter than those with low or no BMI1 expression suggesting that BMI1 is a key regulator and a valuable molecular marker of therapy failure in gastric cancer patients. It also suggest that BMI1 may be regulated by factors other than Mel-18, and that Mel-18 could only partially regulate BMI1, particularly during advanced stages of gastric cancer, which can explain why Mel-18 expression correlated with BMI1 but was not an independent prognosis factor.

Taken together, gastric tumor tissues expressed significantly higher BMI1 and lower Mel-18 compared with normal gastric mucosal tissues, and BMI1 correlated with lymph node metastasis, clinical stages, and prognosis. Hence, detection of Mel-18 and BMI1 expression may be helpful in supporting the diagnosis and determining the prognosis of gastric cancer in clinical practice.

Although *INK4A/ARF *locus is the most cancer relevant target of BMI1 and Mel-18, there are several new reports, which suggest that these PcG proteins, in particular BMI1 may regulate tumorigenesis independent of p16 pathway [17,24,25,30]. Our present data using gastric cancer cells also suggest that BMI1 and Mel-18 can regulate tumorigenesis, cell migration and metastasis at least partially independent of p16, which is a known tumor suppressor involved in regulation of cell proliferation and possibly metastasis [31-33]. Another important target of BMI1 and Mel-18 appears to be AKT [15], which is known to regulate tumorigenesis and cancer metastasis in several cancers [34-36]. Accordingly, our present study suggests that regulation of gastric tumorigenesis and metastasis by Mel-18 and BMI1 may involve AKT, which was overexpressed in gastric cancer tissues and positively correlated with the depth of invasion and lymph node metastasis in gastric cancer patients.

## Conclusions

In summary, our studies suggest that Mel-18 and BMI1 play important but opposite roles in the tumorigenesis, progression and metastasis during gastric cancer development; BMI1 acts as an oncogene, while Mel-18 functions as a tumor suppressor. Additionally, Mel-18 and BMI1 may regulate tumorigenesis, cell migration and cancer metastasis via both p16- and AKT-dependent pathways. Finally our studies suggest that determination of the expression of BMI1 and Mel-18 may help in supporting the diagnosis and determining the prognosis of gastric cancer in clinical practice.

## Abbreviations

PcG: Polycomb group; RNAi: RNA interference; PKB: Protein Kinase B; BMI1i: BMI1 shRNA; Ctrl i: control shRNA; SA-β-gal: Senescence-associated beta galactosidase; tAKT: total AKT; pAKT: phospho-AKT; IHC: Immunohistochemical; HMECs: human mammary epithelial cells.

## Competing interests

The authors declare that they have no competing interests.

## Authors' contributions

XWZ , YPS, QL, WQ, YWL, YFC perform the experiment. BYL, FCZ, and JL supervised the experiment or provide technique support. XWZ, GPD, and WJG analyzed the data and prepared the manuscript. WJG designed the experiment and supervised the project. All authors read and approved the final manuscript.
